# Strength Training in Children: A Systematic Review Study

**DOI:** 10.3390/children12050623

**Published:** 2025-05-12

**Authors:** Borys Bismark León-Reyes, Dilan Galeano-Rojas, Manuel Gámez-Vílchez, Claudio Farias-Valenzuela, Claudio Hinojosa-Torres, Pedro Valdivia-Moral

**Affiliations:** 1 Facultad de Educación, Universidad Estatal de Milagro UNEMI, Machala 070201, Ecuador; bleonr@unemi.edu.ec; 2 Department of Musical, Plastic and Corporal Expression Didactics, Faculty of Educational Sciences, University de Granada, 18071 Granada, Spain; dagaleanor@correo.ugr.es (D.G.-R.); mgamez-vilchez@correo.ugr.es (M.G.-V.); 3 Escuela de Ciencias de la Actividad Física, Universidad de Las Américas, Santiago 9170022, Chile; claudio.farias.valenzuela@edu.udla.cl; 4 Facultad de Educación y Ciencias Sociales, Universidad Andres Bello, Viña del Mar 2520000, Chile; claudio.hinojosa@unab.cl

**Keywords:** training, strength, children, primary education

## Abstract

Background: With the increasing prevalence of sedentary lifestyles and childhood obesity, physical activity and exercise have emerged as essential pillars of health promotion in childhood. In this context, schools play a fundamental role as key players in the implementation of interventions that promote healthy habits and a more physically active life. Objectives: The purpose of the present study was to conduct a systematic review of the benefits of strength training in primary school students. Methods: The review was carried out using the Web of Science and Scopus databases, following the guidelines of the PRISMA statement and a PICO strategy. The studies were selected according to different inclusion and exclusion criteria, resulting in 11 scientific articles published in English during the last 5 years (2020–2024). The methodological quality of the studies was assessed using the STROBE checklist. Results: The results suggest that strength training with elementary school students improves physical and motor performance variables, as well as cognitive, psychosocial and health variables. Likewise, it can be observed that plyometric strength-training methods and unilateral and combined programs with aerobic and motor skills training are highly effective and favor adherence to physical exercise. This also demonstrates the importance and necessity of developing motor coordination skills from an early age, since they represent a determining factor in strength training as the exercises become more specific and complex. Conclusions: In conclusion, implementing strength training programs in primary education confers several benefits at an integral level for the students and is crucial to improving the lifestyle and quality of life of students.

## 1. Introduction

Childhood and adolescence represent critical moments in human development, when important foundations for lifelong health and well-being are laid down [1,2]. During these years, children experience rapid physical, cognitive and social growth, which makes them especially receptive to environmental influences, including physical activity (PA) practices [3]. In this context, physical exercise is presented as a fundamental component to promote health and prevent diseases in childhood and, subsequently, in adulthood [4,5]. It is within this framework that an interest in strength training in elementary school students arises, driven in recent years by growing concern about sedentary lifestyles and childhood obesity, as well as the need to promote active lifestyles from an early age [6,7].

This type of training, commonly associated with adult athletes and bodybuilders, has begun to gain attention within the field of children’s health as a potentially beneficial way to improve children’s physical well-being and development [8,9]. However, the emergence of this trend has also led to questions and concerns about the risks and side effects of introducing strength training at an early age [8,10]. Historically, myths about strength training in childhood have been debated regarding its potential contraindications, in terms of affecting growth, causing injury or interfering with normal motor development [11,12]. However, recent research has brought a new level of knowledge to the topic, demystifying many of these issues and affirming that proper strength training programs in children are safe and beneficial for motor, muscle and bone development [11,13]. Therefore, this is actually a physical quality for which training should begin from the first years of life [14,15].

Among the possible advantages and benefits of strength training, we highlight the related improvements in muscle strength, bone health, body composition and cardiac function, in addition to benefits regarding academic performance, self-esteem and socio-emotional well-being [9,16,17,18]. In this sense, detecting sensitive moments during childhood in which strength exercise benefits components of the child’s physical development reinforces the relevance of implementing this type of measure from an early age [13]. According to Lloyd et al. [17], if a 7- or 8-year-old child is ready to participate in sports activities, he or she can be initiated into a strength training program. This evidence indicates that early childhood is an ideal period in which to carry out strength-training programs in educational settings. Furthermore, the importance of promoting PA in childhood is supported by health organizations and experts in the field. The World Health Organization [19] states that a lack of physical exercise represents a major risk for the development of non-communicable diseases; therefore, this suggests the promotion and implementation of various physical activities, including strength training, which is key to strengthening muscle and bone health.

In this regard, a study conducted by Chulvi et al. [20] argues that regular engagement in strength-training programs should be promoted progressively by gradually moving from bodyweight, medicine ball and resistance band exercises at initial stages, to disc, dumbbell and barbell exercises at more advanced stages, with a view to facilitating and improving adherence to regular PA engagement. This should go hand-in-hand with a systemic approach to athletic and sports engagement, in which muscle strength and power are trained and lay the foundations for facing sporting demands and reducing the risk of muscle injuries and neuromuscular dysfunctions in the short, medium and long term [21,22]. For this reason, it is necessary to have specialist educators in children’s fitness available who can master, in addition to training principles, the contextual specificities inherent to this stage and who are, therefore, able to convey messages assertively, educate about and generate enthusiasm for engagement [20].

In this sense, recent review studies reveal the importance of strength training in children and adolescents from different perspectives, comparing strength training with power training [23], analyzing the effects of resistance on muscle strength [24] and evaluating strength levels in patients with different pathologies [25,26]. Another such study, a systematic review performed by Valle et al. [9], stands out by analyzing the effects of strength training in children with regard to various health parameters, identifying improvements in anthropometric indices and physiological and psychological variables. However, the above studies pertained to various different contexts, which suggests that it would be valuable to further explore the use of strength training in the primary school setting, especially in relation to possible benefits for general student wellbeing. Against this background, the need to deepen the study of strength training in the primary education context is justified, taking into account the problems faced by this population in terms of sedentary lifestyles and the potential benefits of strength training at a comprehensive level, after dismissing myths about the negative impact of this type of training. Such studies can provide relevant information to guide the implementation of strength training programs in schools, promoting a more active lifestyle from an early age [7,8,27]. In this sense, in line with that discussed above, the present research posed the question: “What are the benefits of strength training for elementary school students?”. In order to answer this question, the study proposed a systematic review of the existing scientific literature on strength training in elementary school students in order to identify and analyze its benefits at a comprehensive level.

## 2. Materials and Methods

A systematic review was conducted following the guidelines established in the PRISMA statement [28], to collect and analyze findings related to strength training in the primary education setting, employing the PICO strategy [29].

### 2.1. Procedure

A literature search was performed using the Web of Science (WOS) and Scopus databases. Search terms related to strength training and elementary education were selected. These included the following keywords: “Strength”, “Strength Training”, “Strength Exercise”, “Elementary Education”, “Physical Education” and “Primary Education”. The search strategy consisted of using a combination of search terms with the Boolean operators “OR” and “AND”, establishing the following search equation: “Strength” OR “Strength Training” OR “Strength Exercise” AND “Elementary Education” OR “Physical Education” OR “Primary Education”. To this end, in order to ensure consistency in the selection of articles, the following inclusion and exclusion criteria were defined: articles published in English or Spanish during the last 5 years (2020–2024), with a population of elementary education students (6–12 years), in which the relationships and/or effects of strength training in the child population were analyzed, and in which significant results were achieved. The researchers conducted searches independently, using the previously established search equation, and then compared the results obtained. Each author applied the inclusion and exclusion criteria defined in advance for selecting the articles to be analyzed. Subsequently, the selected papers were shared among all team members for a second evaluation. After a collaborative analysis, the authors reached a consensus on the final selection of the articles, ensuring that the established criteria were respected.

### 2.2. Search Strategy

**Figure 1:**
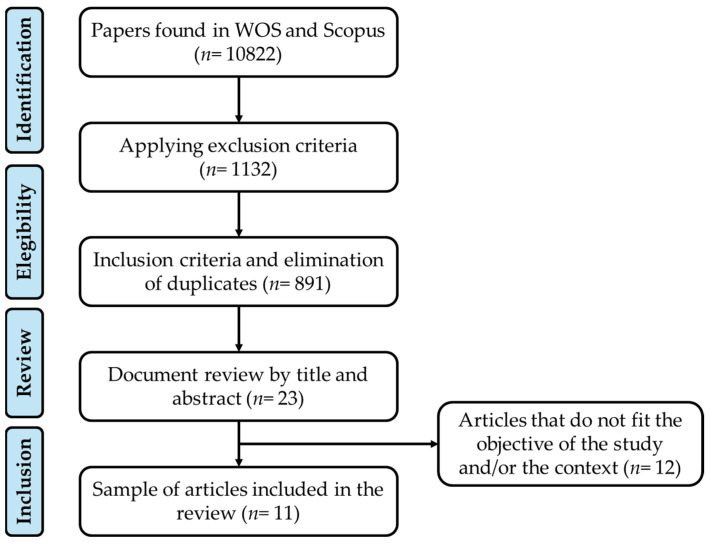
Flow diagram of the systematic review [28].

The search process was divided into several stages. Initially, a preliminary exploration was carried out using various combinations of terms, employing the Boolean operators “OR” and “AND”. This yielded a total of 10,822 articles related to the topic of interest for this review (WOS = 6579 and Scopus = 4243). Subsequently, exclusion criteria were applied, resulting in 1132 articles in the second phase (WOS = 414 and Scopus = 718). In the third filter, inclusion criteria were applied and duplicates were eliminated, reducing the number of studies to 891. Finally, these 891 articles were reviewed using their titles and abstracts, selecting only those that met the objectives of the review. In this way, a total of 23 articles were selected and analyzed in greater depth, ultimately selecting a total of 11 articles for inclusion in the present review (Figure 1).

### 2.3. Methodological Quality

Two investigators independently assessed the quality of each study included in the final review sample and disagreements were resolved by consensus. The STROBE checklist [30] was used, which is composed of 22 items that assess the title and abstract, introduction, methods, results, discussion and other information. Methodological quality ratings were grouped into high (>70%), moderate (50% to 69%) and low (<50%) [31]. In this sense, high methodological quality was attributed to 73% of studies and moderate methodological quality to the remaining 27%. Thus, the included studies were characterized as having a relatively moderate risk of bias (Table 1). Finally, given that the study represented a systematic review, ethical approval was not required. However, it is important to note that all studies included in the present review were approved by the respective ethics committees of their institutions.

## 3. Results

A total of 11 articles were included for which data were presented regarding the authors, year of publication, objectives, design and participants, intervention/variables and instruments, and outcomes (Table 1). All of the selected studies focus on the Primary Education stage and cover the publication period from 2020 to 2024. Most of the included articles were conducted in 2022 (*n* = 4), followed by 2021 (*n* = 3), 2023 (*n* = 2) and only one publication in both 2020 and 2024. In addition, it should be noted that all included studies were written in the English language.

A total of 19,987 individuals participated in the included studies, with the study conducted by Drenowatz et al. [34] having the largest sample, made up of more than 18,000 students. Additionally, it is notable that participants came from different countries: Italy, Tunisia, Chile, Austria, Australia, England, Ukraine, Colombia and Russia. In other words, this review considers data collected from at least one country from each continent.

Furthermore, 27% of studies employed a cross-sectional design (*n* = 3) for descriptive correlational purposes, finding that overweight children have lower motor competence and physical fitness levels [34] and that high levels of PA and strength influence executive and short-term memory functions [32,33]. In contrast, 9% of studies employed a longitudinal approach (*n* = 1), with one study revealing that positive attitudes, healthy anthropometric indices and appropriate levels of activity and fitness could result in short- and long-term (over 35 years) muscle benefits [5]. Finally, 64% of studies were experimental in nature (*n* = 7), with almost all interventions comprising between 8 and 30 sessions that were delivered during physical education classes, with 2–3 sessions per week and sessions lasting 50–60 min. An exception to this is seen in the study conducted by Grainger et al. [36], in which movement skills sessions that either included or omitted strength tasks complemented the usual physical education classes. Also, in the work of Sortwell et al. [39], the experimental group only performed plyometric training during the warm-up (15 min) and achieved more improved motor competence and muscle power in relation to the control group. Similarly, in an intervention based on muscular strength exercises, cardiorespiratory fitness and Tabata [5], and in an intervention targeting physical qualities (strength, speed, flexibility and coordination) [1], the experimental groups also significantly improved their perceptions of general physical fitness and achieved improvements in physical capacities, respectively. In studies with primary school students, conducted by Chaouachi et al. [35], Jaimes et al. [38] and Ivashchenko et al. [37], intervention groups performed unilateral training, multiple jump training, sled towing and uphill running, completing strength training combined with coordination and motor skills, and afterward exhibiting improvements in physical fitness and motor development.

## 4. Discussion

The purpose of this study was to carry out a systematic review of strength training with elementary school students, in order to determine the training’s benefits at a comprehensive level. The relevance of this study lies in the importance that strength training has demonstrated in various populations, both at a physical and motor level, as well as in relation to other cognitive, psychosocial and health components [9,32,33,40], which, in the case of the present research and overcoming the myths related to strength training at an early age, is oriented to strength training. It assesses a population of students in primary education, in order to identify the potential benefits of strength training from a holistic perspective, it being useful for improving the interventions carried out in school, as well as improving the style and quality of life of students at this educational stage [8,27].

Initially, it was observed that all the studies analyzed followed a quantitative research approach, mostly experimental in nature, with, to a lesser extent, a non-experimental study; most are mainly cross-sectional [32,33,34], only including one longitudinal study [6]. In the experimental studies, different types of strength training interventions were performed. Among these, plyometric training [39], unilateral strength training [35], aerobic training, combined with high intensity intervals [5] and multiple jump training, sled pulling and uphill running [38] stand out. Also, in other works, strength training was implicit or combined with other intervention proposals. In the work of Grainger et al. [36], one group applied an experimental fundamental motor skills training program, plus a strength training program. In another study, artistic gymnastics was used in a complementary strength and coordination training program [37], while, finally, the intervention conducted by Polevoy [1] focused on the training of physical qualities in a general way.

In this regard, it should be noted that all these studies evaluated and compared the effect of the interventions on the qualities and indices of physical fitness and motor development, finding significant improvements in most of the variables analyzed. Among the main findings, we highlight the greater gains in upper body strength in the group of children subjected to unilateral strength training (chest press and manual grip) compared to the group of adults [35]. This unilateral training of large muscle groups may produce bilateral upper and lower extremity benefits [35]. However, it should be taken into account that, in the administration of external loads, bilateral exercises may be more advisable due to the supporting and maturation action that they favor, since this exerts a unilateral preventive protective effect, taking into account that, in infantile ages, there is usually no mastery of certain specialized movements in strength training [15]. Hence, this shows the importance of prioritizing the learning of proper technique [41], in line with the adequate training of physical education teachers in the school context to ensure the correct execution of strength exercises, taking into account the particular abilities and needs of students [20,42]. In this way, safe and effective muscle development is promoted, since the specificity of training and neuromuscular adaptations are a priority at this age [20,35].

Similarly, the greater efficacy of the combined program of strength training and fundamental motor skills training on tests of grip strength, agility, jump height and sprint speed is highlighted in comparison with the group that only followed the fundamental motor skills program [36]. This is consistent with the recommendations of Risueño [43] and Le-Cerf et al. [12], who suggest that strength training at these ages should be combined with other types of aerobic activities with a playful approach, as this improves adherence to training, and, in terms of motivation, enjoyment and pleasure, favors the attitude toward and predisposition to practice. This is corroborated in the work of Sepúlveda-Figueroa et al. [5], in which muscular strength exercises, based on the Tabata method, were preceded by cardiorespiratory fitness exercises, achieving improvements in student perceptions of general physical fitness and even affirming that this type of training can also influence academic performance and psychosocial constructs. This may be due to the fact that satisfying the need for competence leads to higher rates of effort and commitment, which, subsequently, improve motivation and lead to better outcomes [44,45].

In this same line, we considered the study by Polevoy [1], in which a pedagogical experiment based on the development of various physical qualities improved the effectiveness of training in children aged 9 to 11 years, highlighting the importance of complementing the physical education class with a comprehensive physical preparation program, due to its value and potential in terms of student health and welfare. This is in line with the motor competence model, since the ability to integrate knowledge, attitudes and emotions in order to interact assertively with one’s environment is based on the harmonious development of the physical capacities of strength, endurance, speed, flexibility and coordination [46,47,48].

Next, the research of Ivashchenko et al. [37] stands out; they evaluated the effectiveness of various methods of strength training for the proper development of motor skills, identifying that the main method of strength development has previously been the combined method, ordering the exercises progressively according to dynamic effort, maximum effort, isometric effort and repeated effort, so that there is a learning effect of motor skills combining the development of strength and coordination, taking into account that these more complex exercises require more training time. Thus, the development of motor coordination skills at this age is crucial for achieving optimal control, coordination and the regulation of body movements, especially in relation to strength training, as a means of preventing injuries and ensuring energy economy and efficient muscular effort [49,50]. Indeed, greater motor competence also means a greater motor repertoire [48]. That is, as the development of motor skills is perfected, one is able to execute a greater number of movements and actions more fluently, precisely and efficiently in response to environmental and task demands. In an integrated neuromuscular approach, trainers combine different methodologies to facilitate the acquisition of skills and improve physical condition; in this way, they boost motivation toward engagement and reduce the level of boredom that is common at these ages [41,48].

In contrast, among the main findings, the clinical importance of the sled drag and uphill running training methods to improve physical performance are also highlighted, compared to the multiple jump method, despite the fact that the latter also proved to be effective [38], since previous studies show that it may have less effect in those students with no previous training experience [51,52]. However, these are results that should be interpreted with caution since, as stated by Martínez-Pérez and Vaquero-Cristóbal [53] and Risueño [43], this type of strength training (multiple jumps) at early ages turns out to be among the most effective and accessible method, since it is developed using self-loads and produces significant gains. This is consistent with the work of Sortwell [39], in which plyometric-based training during warm-ups with children aged 7 to 8 years generated significant increases in motor competence and muscular power in the upper and lower body. This is in line with the findings of the review studies by Balado and Diaz [7] and Le-Cerf et al. [12], which corroborate the trends, usefulness and adaptability of plyometric training with children and young people, highlighting its benefits in relation to physical fitness, motor coordination and the biomechanics of movement, ruling out possible harmful contraindications, provided that the intensity, frequency and volume of training are adjusted to the needs and abilities of the students.

Finally, in relation to the experimental studies presented, there are a number of consistencies with the systematic review published by Valle et al. [9]. Firstly, the number of sets per exercise ranged between two and three, with between 8 and 20 repetitions, depending on the nature of the exercise and the training, which ranged between high and moderate intensities. Secondly, the rests between sets and exercises lasted between one and two minutes; thirdly, interventions comprised between 8 and 30 training sessions that were performed two or three times a week on non-consecutive days. This, in turn, concurs with the findings of Peinado-Rincón et al. [41], who argue that the appropriate intensity for children aged 8 to 13 years is between 60% and 80% 1 RM or, in certain cases, even lower, provided that technical execution is prioritized. In addition, in most of the works mentioned, two to four sets of 6 to 15 repetitions per exercise were also performed, with higher volumes being performed at lower intensities and vice versa. In the same way, rests should last between 1 and 2 min for moderate intensity exercises, in accordance with the principle of individualization, which can lead to longer rests in particular cases. For this reason, a training frequency of two to three times per week is optimal, including at least one day of rest between sessions, as stated by most studies in their respective interventions.

However, the studies also included those that did not perform interventions, which were focused on analyzing the association of strength with different variables of physical fitness, cognitive capacities and health. The works of Agostino et al. [32] and Contreras-Osorio et al. [33] focused on examining the association of strength with cognitive abilities. On the one hand, it was observed that strength levels can positively influence short-term memory capacity in elementary school students, mainly in those of older age, as a product of the functional and structural adaptations induced by brain plasticity in different areas of the brain, favoring cognitive functioning, executive function, attention and even academic performance [32,54,55]. On the other hand, in the study by Contreras-Osorio et al. [33], several relationships were found between anthropometric factors, executive function, activity and physical fitness, highlighting, in relation to strength, that greater muscular power of the lower and upper body is linked to greater cognitive flexibility, inhibition, planning, attention and working memory.

These findings are consistent with previous research that has documented similar results in different population groups [16,56]. Furthermore, the study by Valle et al. [9] also indicates that a combination of strength training and aerobic training can lead to improvements in students’ cognition. In this regard, De Greeff et al. [55] emphasize that, to maximize the benefits of this type of training, it is essential that the proposed activities maintain a high level of cognitive and attentional engagement, following an adequate dosage to avoid overstimulation.

In the work of Drenowatz et al. [34], after analyzing the relationship between physical capacities as a function of height and weight, the authors found that although overweight students showed lower motor competence and physical fitness, they achieved better results in those upper body strength tests in which external loads were used, and not in those exercises using their own body weight. Moreover, these results over time tended to be better in those students of normal weight. This is in line with the findings and conclusions of the longitudinal study by Fraser et al. [6], which affirms the need to generate strategies aimed at increasing muscle strength at an early age, due to its multiple benefits in terms of health, well-being and quality of life in the short and long term, generating positive attitudes about the importance of physical exercise, fitness and body image. In short, to promote appropriate learning and training experiences, which allow for achieving, as shown in this review work, numerous benefits related to motor competence, physical fitness, health and cognitive functioning, one should include the various possibilities of interventions that fit the interests and needs of students in the school environment, giving special importance to the subject of physical education as an appropriate setting in which to promote this type of strength development practice in children, in order to improve the style and quality of life of students [6,32,33,57].

### Limitations and Future Perspectives

The present review has some limitations that should be taken into consideration. First, the heterogeneity of the study designs and of the methodologies employed makes direct comparison of the findings difficult and may impinge on their generalizability. Coincidentally, in the present review, only cross-sectional studies analyzed the associations of strength training with cognitive variables, which means that data should be interpreted with caution as causal relationships cannot be established. Nevertheless, according to the methodological quality criteria that were evaluated, all the studies were of high (8) and moderate (3) quality; thus, the risk of bias was considered to be relatively moderate.

However, the variability in the ages of the participants and the duration and intensity of the training programs, as well as the geographic, sociocultural and educational settings, may affect the overall analysis of the results. In addition, most of the studies reviewed are of short to medium duration, which precludes assessing the long-term impact of these interventions. Therefore, in future research, we suggest the design of more homogeneous and longitudinal studies that allow a more accurate and generalizable evaluation of the effects of strength training in primary school children. Also, it would be beneficial to explore how different variables, such as gender and socioeconomic level, can modify the effects of this type of training. Finally, it is recommended to develop and implement strength training programs, integrated within the physical education curriculum, which not only focus on physical benefits, but also promote cognitive and emotional development, thereby improving opportunities for access and participation at the school level, adapting such interventions to the individual and collective needs of students.

## 5. Conclusions

In response to the research questions and objectives of this review, it is concluded that: strength training in elementary school students not only improves physical and motor performance variables, but also contributes to the development of cognitive, psychosocial and health factors, representing an important means to improve the quality of life of students at an integral level. In turn, we conclude and corroborate the effectiveness of the different methods of strength training, including plyometric training, unilateral strength training and combined programs that integrate aerobic components and motor skills in a playful way. This playfulness represents one of the most outstandingly successful techniques since it favors motivation to continue practice and adherence to physical exercise, positively influencing multiple factors at the academic level and improving the integral well-being of students, with the potential for such habits to be prolonged in time until adulthood.

Likewise, the need to develop motor coordination skills from childhood is highlighted. This is important in order to boost motor competence and, as a result, the motor repertoire that forms the basis of adequate technique development for the performance of strength training exercises. This is also required in order to prevent injuries, generate efficient motor and muscular resolution and guarantee economy of effort. Teaching such skills should be aligned with teacher training to promote safe and efficient muscular development that is adapted to student capacities and needs.

With regards to the methodological characteristics of strength training interventions in the primary education setting, it is concluded that frequency, volume, intensity and rest parameters should be adjusted to match training characteristics. A range of 8 to 30 sessions is recommended, with sessions being composed of two to three sets comprising 8 to 20 repetitions, as a function of the specific training objective. Intensities should be moderate to high, with 1- to 2-min breaks between sets and exercises. That being said, certain contextual particularities necessitating adaptations to individual needs must be borne in mind.

In short, this review shows the importance of integrating strength training programs at the primary school stage, taking into account the individual and collective needs of students in order to maximize the programs’ potential benefits, not only for physical and motor development, but also for their contributions in terms of well-being, cognitive development and quality of life.

## Figures and Tables

**Table 1:** Characteristics of the selected studies.

Authors and Year	Objectives	Design and Participants/ Country/Methodological Quality	Intervention/ Variables and Instruments	Results
Agostino et al. [32]	Examine whether physical strength can positively influence short-term memory efficiency in elementary school children. Furthermore, in the case that an effect exists, determine whether age plays a role in mediating and moderating this influence.	Design: Cross-sectional and exploratory. Sample: 75 students (45 boys and 30 girls) from an elementary school (from 2nd to 5th grade), with a mean age of 8 ± 1 year. Country: Italy Quality: High	Physical strength: Overhead (backward) medicine ball throwing test. Efficacy of short-term memory: Digit span test from the Wechsler Intelligence Scale for Children (WISC)	Physical strength positively affects short-term memory, with age moderating but not mediating this effect. That is, age potentiates the positive impact of physical strength on short-term memory in older students.
Contreras-Osorio et al. [33]	Evaluate the associations between anthropometric parameters, PA, physical fitness and executive functions among elementary school children who returned to school after COVID-19 confinement in Chile.	Design: Cross-sectional. Sample: 90 students (46 boys and 44 girls) in 5th and 6th grade, with a mean age of 11.45 ± 0.68 years. Country: Chile Quality: Moderate	Anthropometric parameters PA: PAQ-C Physical fitness: Standing long jump test (SLJ), 10 × 5 m agility shuttle run test, handgrip strength and the six-minute walk test. Executive function: Neuropsychological Assessment for Executive Function in Children Battery	Higher levels of PA, sprint performance, lower body muscle power and greater upper body strength were associated with better working memory, cognitive flexibility, inhibition, attention and planning.
Drenowatz et al. [34]	Provide objective information on study and fitness assessments, and on differences according to the age and weight status of elementary school children in Upper Austria.	Design: Transversal. Sample: 18,168 students (53% children) from 2nd, 3rd and 4th grades of primary school, aged between 6 and 11 years old. Country: Austria Quality: Moderate	Anthropometric measurements; cardiorespiratory endurance: 6-min run; muscular strength and power: medicine ball throw and countermovement jump; speed: 10 m sprint and tapping test; agility: obstacle course; flexibility: standing upright test	Overweight children showed lower motor competence and physical fitness, except in upper body strength. In addition, improvements in test performance with age were less noticeable in overweight children compared to their normal-weight peers.
Fraser et al. [6]	Determine the childhood factors associated with predetermined muscle strength trajectories across the lifespan.	Design: Prospective, longitudinal (three time points between childhood and adulthood). Sample: 1280 schoolchildren aged 7 to 15 years. Country: Australia Quality: High	Anthropometric measurements; sociodemographic characteristics; elements of health and risk motivation; muscle power: longitudinal jump; cardiorespiratory fitness: running/walking 1.6 km; flexibility: the sit and reach test; muscle strength: right- and left-hand grip strength, shoulder flexion and extension and leg strength with dynamometers	PA, physical fitness, positive attitudes toward health and a healthy weight in childhood imply muscular benefits in the short term and can lead to better muscle strength in the long term.
Chaouachi et al. [35]	Compare the effects of global training and cross-training in adults and young adults performing unilateral isoinertial chest press vs. an isometric grip on contralateral and ipsilateral limb tests.	Design: Mixed-model and experimental. Sample: 89 children (10–13 years) and adults (18–22 years), divided into groups performing: unilateral chest press training; grip strength training; and CG (15 adults in each group). Country: Tunisia Quality: High	8 weeks, 3 times per week. In the EGs, in each session, the series of exercises was performed until momentary failure, with a gradual progression that varied the number of repetitions and series performed each week	Strength gains following training were significantly greater in adults than in young participants when assessed via lower body testing, whilst youth training gains outperformed adults in upper body testing. Training specificity is crucial, and neuromuscular adaptation is more relevant in youth. Suggests the need for training strategies and adaptations between age groups.
			Pre- and post-test measurements of dominant and non-dominant limbs, unilateral chest press and leg press at 1 rep maximum (1 RM), hand grip, knee extension and flexion, and maximal voluntary isometric contractions (MVIC) of elbow extension and flexion	
Grainger et al. [36]	Evaluate the effect of fundamental movement skills (FMS) in isolation or in combination with a strength training intervention on children’s fundamental movement skills.	Design: Exploratory pilot study. Sample: 59 students (53% girls) aged 10 to 11 years from three primary schools. FMS (*n* = 18), FMS + Force (*n* = 20) and control (*n* = 21) groups. Country: England Quality: High	Intervention based on FMS and FMS + Strength: 8 sessions, in addition to PE classes, lasting 50–60 min, with 5 activities per session. The FMS + Strength group included extension and flexion exercises of the hip, knee and ankle joints during the last 2 activities (15 min, respectively) of each session (sets of 4 × 10)	Possible positive effects of the integrated combination of fundamental movement skills and strength training as a tool to develop fundamental movement skills in children are demonstrated.
			Pre- and post-test measures. Fundamental movement skills: Canadian agility and movement skills assessment; locomotor performance: grip strength, agility, countermovement jumps and sprints (40 m)	
Ivashchenko et al. [37]	Determine the relationship between teaching media and motor training in elementary school PE classes.	Design: 2 × 2 full factorial experiment. Sample: 32 children who were 8 years old at the beginning of the experiment. Four groups comprising eight students each. Country: Ukraine Quality: Moderate	The intervention method for each group was as follows. 1: Basic motor teaching; 2: coordination training, with basic motor teaching techniques; 3: strength training, along with basic motor teaching; 4: strength and coordination training, in addition to motor actions	Strength and coordination of movement is considered a condition for successful motor skill development. As an exercise becomes more complicated, the time allocated to strength and coordination training increases. A holistic approach is advocated that makes room in PE classes for these three aspects.
			Pre- and post-test measurements of unidirectional and multidirectional coordination exercises, errors in temporal accuracy, errors in muscular effort evaluation and errors in vestibular stability	
Jaimes et al. [38]	Identify the effects of three strength training methods during an eight-week training program on speed, vertical jump height, and speed/agility in prepubertal schoolchildren.	Design: Quasi-experimental. Sample: 63 prepubertal children with a mean age of 9.2 ± 0.5 years. Participants were randomly assigned to one of three training groups: multiple jumps (*n* = 20), sled towing (*n* = 21) and uphill running (*n* = 22). Country: Colombia Quality: High	8 weeks, 3 sessions each week, of: Multiple jumps: 2 to 5 sets, comprising 8–20 reps of bounding, forward hopping, hurdle jumps and box jumps; sled tow: 1 to 3 sets of 2, comprising 10 reps towing 2.5 to 10% of bodyweight over 5, 10, 15 or 20 m (depending on the exercise); uphill run: 1 to 4 sets, comprising 2 to 8 reps of running 6 to 26 m uphill (depending on the exercise)	Statistically significant changes in all variables were found for each group with different magnitudes of effect. All three programs improve jumping and speed/agility performance, but based on clinical significance, the uphill running and sled dragging methods can be considered more efficient in improving physical performance in children.
			Pre- and post-test measurements of vertical jump (ABK, CMJ, SJ), standing long jump (SBJ), 4 × 10 m round-trip running test and sprint test (20 m)	
Polevoy [1]	Increase the effectiveness of the training process with athletes from 9 to 11 years of age, based on the development of certain motor skills.	Design: Pedagogical experiment. Sample: 50 children aged 9 to 11 years old assigned to an EG (*n* = 25) or a CG (*n* = 25). Country: Russia Quality: High	10 weeks, 3 sessions each week. In each session, 6–7 physical skill exercises were performed during the main section (15 min) of the sessions. Exercises targeted: Speed: 3–4 sets of 8–10 reps; strength: 3–4 sets of 10–20 reps; flexibility: 2–4 sets of 10–12 reps; coordination: distances of 15–20 m; 1–2 min rest for each exercise	The EG exceeded the CG for almost all indicators. The pedagogical experiment positively impacted physical skill development in younger athletes and significantly increased the efficiency of the training process.
			Pre- and post-test measurements of motor skills. Leg power: longitudinal jump; speed and agility: shuttle run; leg strength and speed: squats (30 s); hand strength: dynamometer; and flexibility: sit and reach test	
Sepúlveda- Figueroa et al. [5]	Analyze the effect of a PA program on perceptions of physical fitness in schoolchildren aged 9 to 11 years.	Design: Quasi-experimental. Sample: 20 students (14 boys and 6 girls) in the 4th and 5th grades of primary school with a mean age of 10.45 ± 0.89 years, divided into EG (*n* = 10) and CG (*n* = 10). Country: Chile Quality: High	12 weeks, 2 classes per week. In each session in the central part (30 min) nine cardiorespiratory fitness exercises, nine muscular strength exercises (2 sets of 15 × 15) and eight Tabata exercises (2 sets 20 s × 15 s) were performed	The PA program had a positive effect on the perception of general physical fitness and in relation to cardiorespiratory and muscular strength components, with significant differences between groups in favor of the EG.
			Pre- and post-test measures of fitness, cardiorespiratory and muscular strength perceptions: International Physical Fitness Scale	
Sortwell et al. [39]	Examine the effects of plyometric training integrated into PE classes on motor skill performance, a reactive strength index, and upper and lower body muscle power measures in elementary school students.	Design: Quasi-experimental. Sample: 61 students (29 girls and 32 boys) in the 2nd grade of primary school, aged from 7 to 8 years old. Students came from two PE classes. A plyometric group (*n* = 31) and a comparative group (*n* = 30) were assigned. Country: Australia Quality: High	8 weeks, two 50-min classes per week. The EG performed the plyometric-based program as a warm-up (15 min). This consisted of a circuit of stations with upper and lower body exercises, divided into three phases with progressively increasing intensity and decreasing reps (from 2 × 10 to 2 × 6 sets at the end of the intervention)	Significant increases found in motor competence and muscle power of the upper and lower body in the plyometric group versus the comparison group. Inclusion of a program based on plyometric exercises in the warm-up phase of physical education classes can improve motor skills and muscle power in students.
			Pre- and post-test measurements. Anthropometric indices; motor performance: FMS-Polygon; upper and lower body muscle power: medicine ball throw and squat jump test; reactive strength index: drop jump	

CG = control group; EG = experimental group; PE = physical education.

## Data Availability

Data analyzed for this systematic review were taken from the published manuscripts appraised in the manuscript. These studies and data are available on electronic databases or from the respective journal websites.
